# Light heterogeneity affects understory plant species richness in temperate forests supporting the heterogeneity–diversity hypothesis

**DOI:** 10.1002/ece3.8534

**Published:** 2022-02-19

**Authors:** Jan Helbach, Julian Frey, Christian Messier, Martin Mörsdorf, Michael Scherer‐Lorenzen

**Affiliations:** ^1^ Geobotany, Faculty of Biology University of Freiburg Freiburg Germany; ^2^ Chair of Remote Sensing and Landscape Information Systems Faculty of Environment and Natural Resources University of Freiburg Freiburg Germany; ^3^ CEF, ISFORT Université du Québec en Outaouais et à Montréal Montréal Canada; ^4^ Present address: Chair of Forest Growth Faculty of Environment and Natural Resources University of Freiburg Freiburg Germany

**Keywords:** coexistence, community ecology, ConFoBi, environmental heterogeneity, niche dimension, stand structural diversity

## Abstract

One of the most important drivers for the coexistence of plant species is the resource heterogeneity of a certain environment, and several studies in different ecosystems have supported this resource heterogeneity–diversity hypothesis. However, to date, only a few studies have measured heterogeneity of light and soil resources below forest canopies to investigate their influence on understory plant species richness. Here, we aim to determine (1) the influence of forest stand structural complexity on the heterogeneity of light and soil resources below the forest canopy and (2) whether heterogeneity of resources increases understory plant species richness. Measures of stand structural complexity were obtained through inventories and remote sensing techniques in 135 1‐ha study plots of temperate forests, established along a gradient of forest structural complexity. We measured light intensity and soil chemical properties on six 25 m² subplots on each of these 135 plots and surveyed understory vegetation. We calculated the coefficient of variation of light and soil parameters to obtain measures of resource heterogeneity and determined understory plant species richness at plot level. Spatial heterogeneity of light and of soil pH increased with higher stand structural complexity, although heterogeneity of soil pH did not increase in conditions of generally high levels of light availability. Increasing light heterogeneity was also associated with increasing understory plant species richness. However, light heterogeneity had no such effects in conditions where soil resource heterogeneity (variation in soil C:N ratios) was low. Our results support the resource heterogeneity–diversity hypothesis for temperate forest understory at the stand scale. Our results also highlight the importance of interaction effects between the heterogeneity of both light and soil resources in determining plant species richness.

## Graphical abstract

One of the most important drivers of the coexistence of species is the resource heterogeneity of a certain environment. We measured forest structural complexity, vegetation, and resources on forest floor to test whether stand structure has an effect on the distribution of resources, and if so, whether it influences species richness. Our study revealed that forest structure creates a heterogeneous light and soil‐resource environment which then increases species richness.

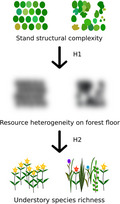

## 1 INTRODUCTION

### 1.1 Environmental heterogeneity hypothesis

It is widely accepted that species with the exact same habitat requirements cannot coexist (Hardin, 1960; Tilman, 1982). However, the large number of autotrophic plant species that do coexist in certain ecosystems, and that rely on the same limited set of abiotic resources (light, water, nutrients, space), seems to contradict this theory. Hence, many ecologists were preoccupied with this contradiction (Angert et al., 2009; Chesson, 2000; Kraft et al., 2015; Tokeshi, 2009; Wright, 2002). Some argued that competitive exclusion may be decreased by top‐down (e.g., herbivory, which controls prevalence of competitive species, Wilkinson & Sherratt, 2016) or bottom‐up (limitation of resources, Wilkinson & Sherratt, 2016) control mechanisms, or other disturbance events (e.g., mowing on meadows, avalanches in alpine habitats, Connell, 1978). Others have suggested alternative mechanisms of coexistence. Competitive exclusion may, for example, be avoided due to environmental heterogeneity within habitats, which creates niches that are differently utilized by various species (Hutchinson, 1957; MacArthur & Levins, 1967; MacArthur & MacArthur, 1961; Sedio & Ostling, 2013; Silvertown, 2004). This “heterogeneity–diversity relationship” as a promoter of species coexistence, and hence a driver of species diversity, is now a widely accepted theory (Chesson, 2000, 2018; Stein et al., 2014; Tews et al., 2004). However, one study investigated the relationship between habitat heterogeneity and birds and found a humped‐back relationship between habitat heterogeneity and bird species richness, deriving the “area‐heterogeneity‐trade‐off theory” (AHTO, Allouche et al., 2012). This theory suggests that the relationship of heterogeneity to species richness cannot be uniformly positive. When heterogeneity in a given area continues to increase, the size of fragments which are characterized by certain conditions will diminish. Consequently, in smaller fragments, it is more difficult for species to survive. Hence, species richness decreases and an unimodal relationship can be assumed. Findings from a meta‐analysis by Heidrich et al. (2020) supported the AHTO for 21% of the tested organism groups in their study. 25% of species groups even showed a monotonous decrease of richness with higher heterogeneity, but for the majority of species (>50%), a monotonous increase was shown. The responsible mechanisms behind such opposing heterogeneity–diversity relationships for different organism groups remain largely unclear. Study outcomes may depend on several contexts, such as the ecosystem type investigated and the spatial scales used to assess diversity (Ben‐Hur & Kadmon, 2020).

Many studies that tested the effects of environmental heterogeneity on biodiversity have investigated open terrestrial habitats (Bergholz et al., 2017; Lundholm, 2009; Morzaria‐Luna et al., 2004), or focused on environmental heterogeneity at landscape scale (see studies in Hammill et al., 2018; Stein et al., 2014). However, forest habitats for instance can show very high within‐habitat heterogeneity at small spatial scales, due to the complex canopy architecture of trees. Further, studies in forests have included other elements of resource heterogeneity, such as vegetation structure, dead wood occurrence, management regimes, wind throws and other disturbances, differences in overstory species richness, or abiotic conditions as proxies for heterogeneity, and many different groups of organism ranging from birds to plants as biodiversity variables (e.g. Bartels & Chen, 2010; MacArthur & MacArthur, 1961; Richard et al., 2000; Stein et al., 2014; Taboada et al., 2008; Tamme et al., 2010). The effect of environmental heterogeneity on diversity will also depend on the spatial scale investigated, which could range from centimeters, meters (i.e., patch scale, e.g., safe sites for seed germination, or sun flecks in the forest understory) to kilometers (i.e., landscape scale, e.g., altitude and climatic belts), depending on the ecosystem of interest. Especially forests, and their small‐scale environmental heterogeneity in the below canopy area, are very little researched with that regard.

### 1.2 Forest structure and resource availability

Even‐aged, monospecific tree stands usually have a rather homogeneous three‐dimensional canopy structure, which might imply a low‐resource heterogeneity for plants that grow below the tree canopy (Fedrowitz et al., 2014). In Central Europe, recent management has shifted from production forestry, with predominantly clearcutting, to near‐natural multifunctional forestry, including retention forestry and the establishment of mixed stands (Gustafsson et al., 2020). This management regime affects light quantity (Forrester et al., 2018), heterogeneity, and consequently understory plant species composition and density (Bengtsson et al., 2000; Duguid & Ashton, 2013; Getzin et al., 2012). Different tree species also have different crown structures (Ampoorter et al., 2015, 2016) and respond differently to disturbances, such as windthrow and snowbreakage, or pest outbreaks (Burton et al., 2014; Hilmers et al., 2018). Understory plant species composition and density, species richness, and functional diversity have been shown to be affected by such retention measures (Halpern et al., 2012; Lindenmayer et al., 2012), partly due to the alteration of microclimate and resource availability across spatial and temporal scales (Aubry et al., 2009; Gustafsson et al., 2012, 2020; Kriebitzsch et al., 2013; Wagner et al., 2011). Enhancing forest structural complexity by creating gaps will not only affect light quality and quantity, but also wind speed and air humidity, soil temperature and moisture, litter input, and hence nutrient availability at the forest floor (Abd Latif & Blackburn, 2010; Lindh et al., 2011). In addition, these abiotic conditions will vary across several temporal scales (daily fluctuations, seasonal changes, year‐to‐year variation, Leuschner et al., 2017). In particular, the spatio‐temporal variability of light quantity at the forest floor is usually greater in structurally more complex forest stands (Liira et al., 2007). Thus, the complex interplay of these changes results in altered resource availability for plants (light, nutrients, water), providing possible advantages to certain species which are differentiated in their adaptation to the respective resource.

### 1.3 Aim of the study and hypotheses

The aims of this study were (1) to investigate whether stand structural complexity of forests induces heterogeneity in light and soil resources below canopy, at a scale of <1 ha and (2) to assess whether heterogeneity of light and soil resources affects understory plant species richness. So far, studies that have investigated the habitat–heterogeneity hypothesis for forest understory plant species actually have rarely tested whether species richness increases when resources are heterogeneously distributed on a scale smaller than 1 ha (Dormann et al., 2020; Reich et al., 2012; Su et al., 2019). The outcome of this study will help to understand if forest structure has an effect on the distribution of resources and if heterogeneous distribution of resources at forest floor increases species richness of the understory.

**FIGURE 1:**
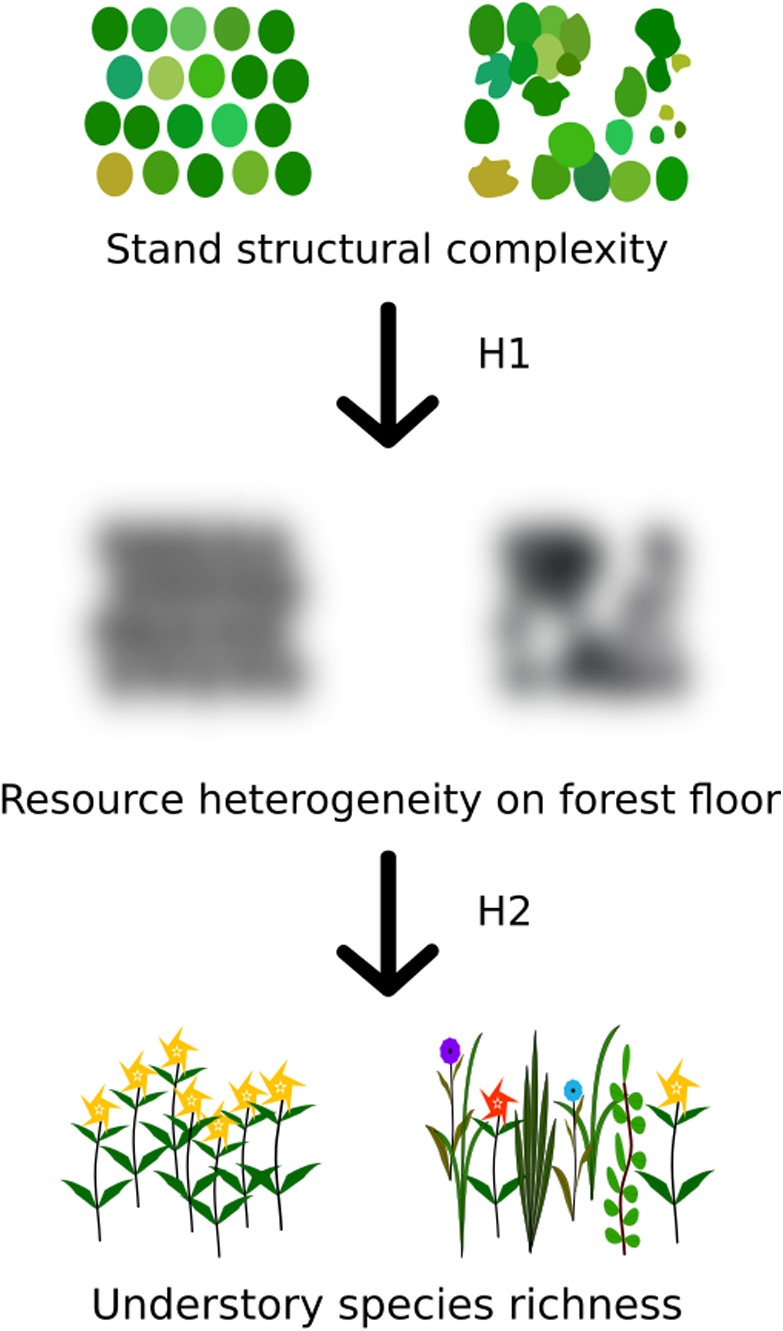
Underlying hypotheses of the study design. See text for the specific hypotheses (H)

We hypothesize that (H1) an increase in stand structural complexity results in an increase in light and soil resource heterogeneity and that (H2) understory plant diversity increases with increasing resource heterogeneity (Figure 1).

To test these hypotheses, we determined understory plant species richness and analyzed the availability of light and soil nutrient resources and their heterogeneity in temperate forest stands along a gradient of stand structural complexity, which had been created through different management interventions in the past.

## 2 MATERIAL AND METHODS

### 2.1 Study site

Field sampling was carried out in the Central and Southern Black Forest, a region in southwest Germany.

Forests in this region are dominated by *Picea abies* (Norway spruce), *Fagus sylvatica* (European beech), *Abies alba* (silver fir), *Acer pseudoplatanus* (sycamore maple), and *Pinus sylvestris* (Scots pine) growing on cambisol, umbrisol, and podzoles (State Office for Geology, Resources and Mining, Baden‐Württemberg, Germany, maps.lgrb‐bw.de/ accessed 2017/2/17)). The forest stands are mostly managed with continuous cover forestry using shelterwood, strip cutting, and single tree selection (“Plenter” forests, Gustafsson et al., 2020) resulting in uneven‐aged forest stands. Some of the investigated stands are monocultures but most are mixed species forests. All stands are more than 60 years old. The bedrock consists of gneiss and granite in the west, with lower Triassic sandstone and middle and upper Triassic limestones toward the east (State Office for Geology, Resources and Mining, Baden‐Württemberg, Germany, maps.lgrb‐bw.de/ accessed 2017/2/17)). The annual average temperature of the region is about 6.9 °C with a yearly average precipitation of 1205 mm (climate station of the city of Titisee‐Neustadt, 846 m asl., www.climate‐data.org, accessed 2020/2/16).

### 2.2 Study design and field sampling

Our study plots belong to the “Conservation of forest biodiversity in multiple‐use landscapes of Central Europe” research project (Storch et al., 2020, Figure 2) and are all located in mountainous temperate forests between 434 m and 1334 m above sea level. A total of 135 plots were selected along a landscape and forest structure gradient. However, we only used the structural gradient for our study, which was determined based on the number of standing dead trees (with three categories: 0, 1–9, >10 trees per ha) assessed from aerial images (State Agency of Spatial Information and Rural Development of Baden‐Württemberg, accessed 2016). Each plot measured 1 ha (100 m × 100 m) in size with a minimum distance of 750 m between plots. Detailed information on study design, plot selection, and categorization of stand structure can be found in Storch et al. (2020).

**FIGURE 2:**
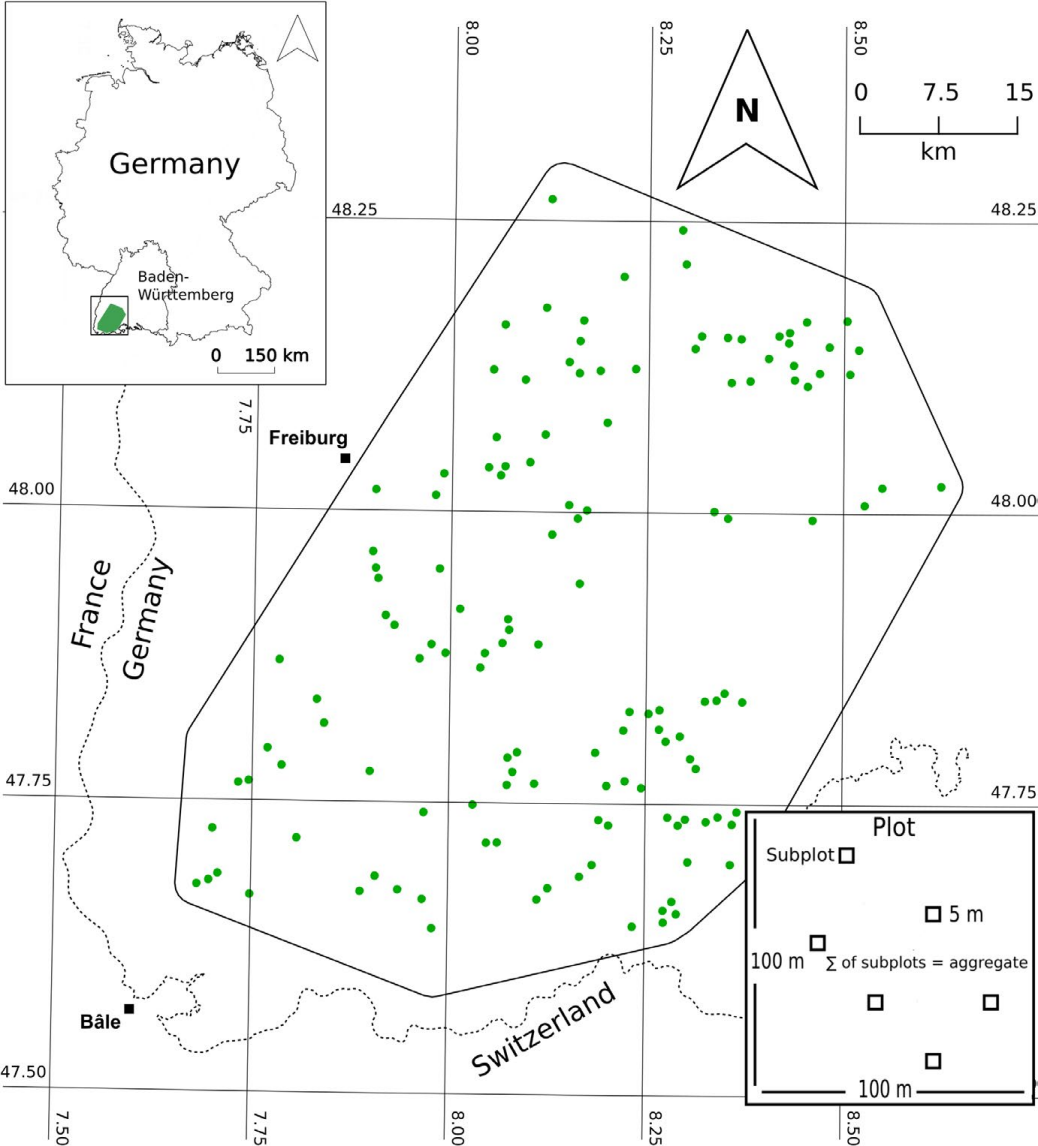
Study sites of our field survey. Figure adapted from Frey et al. (2018). Inset figure on right corner shows the design of the survey

The field sampling took place during the growing season (May–October), between August 2016 and July 2018. To assess plant communities within plots, we established six 5 m × 5 m subplots located within each plot as a predefined grid representative of the whole plot (Figure 2). For each subplot, the cover of each species was visually recorded as one of three categories:

(i) 1%–5% cover or <15 individuals
(ii) 6%–25% cover or more than 15 individuals present
(iii) 26%–100% cover.

Cover estimates were transformed to 2.5%, 15%, and 35%, respectively. We then separated the cover into two height strata: the herb layer comprised cover of all vascular plants including herbal and woody species smaller than 1 m in height, and the tree layer comprised all species taller than 5 m (the shrub layer including all species between 1 m and 5 m was not used in this analysis). We used averages of species cover data within subplots to identify forest plant communities. In addition to the overall presence of vascular plant species within subplots, we screened the whole 1‐ha plot for species that were not within subplots. Plot level species richness was based on the combined species list from subplots and the additional screening. Species names follow the nomenclature of Jäger (2017).

### 2.3 Soil analyses

Immediately after the recording plant cover information, we extracted soil cores within each subplot. Three soil cores of 1 cm diameter and 15 cm depth were taken 1 m from the subplots center. Soil cores were spread at equal distances to each other, defined by a 120° angle from the subplot center. The three soil cores were afterwards mixed together. Soil samples were sieved to a minimum of 2‐mm grain size. To determine water content, 5 g of soil was dried for 48 h at 105°C. Ammonium and nitrate concentrations were measured in fresh soil extracts. We shook 10 g of fresh soil with 25 ml of 1 mol KCl solution for the duration of 30 min. In the case of shallow soil, analyses were made with less soil, keeping the soil:solution ratio constant to avoid differences in extraction strength. If there was too little soil to keep the soil:solution ratio constant, a correction factor was empirically determined to account for the higher extraction strength. For a few plots, there were no fine soil particles (i.e. all particles had a grain size >2 mm). We measured the pH‐value of filtrates using a pH‐electrode (719 S Titrino, Metrohm, Switzerland). With the remaining solution, we measured ammonium and nitrate concentrations, using a microplate reader (Synergy mx, Biotek Instruments, Germany). We determined ammonium colorometrically according to Baethgen and Alley (1989) and nitrate according to Miranda et al. (2001). Concentrations of both compounds were expressed as ppm of dry soil.

We analyzed concentrations of 20 cations and phosphorous using an inductively coupled plasma spectrometer (ICP‐OES, Spectroblue Ti, Spectro Analytical Instruments GmbH, Germany). As standards, we used a 20‐cation solution (ICP multielement standard solution IV Merk KgaA, Germany) and a P solution (Single‐element ICP standard solution, phosphorus, Carl Roth GmbH + Co. KG, Germany). Only those cations known to be macro‐ or micro‐nutrients for plants were further considered (Ca, Fe, K, Mg, Na, and P). To achieve this, we extracted cations from 5 g of fresh soil with Mehlich 1 solution (Mehlich, 1953). Samples exceeding the determination threshold were diluted and remeasured. All concentrations were expressed as ppm of dry soil. Soil C and N concentrations were determined by dry combustion. We dried an aliquot of soil at 60 °C and ground it with a pebble mill for 1.5 min. 50 µg were weighed in tin capsules and measured using a CN analyzer (Vario EL cube, Elementar, Germany). These concentrations were expressed in percentage of dry soil.

### 2.4 Light analysis

We determined the light environment at the subplot level using hemispheric photography. Photographs were taken with a Nikon D90 camera, which was equipped with a Sigma 4,5 mm F2,8 EX DC HSM circular fisheye‐lens. The camera was placed in the center of the subplot, 1 m above the forest floor, with the camera lens facing north and then being leveled horizontally. The hemispheric photographs were taken between May and June 2018, one year after the major plant survey. Missing values in these data were caused by tree removal through logging on the plot or the absence of plot markings. The photographs were prepared with an image processing software (Darktable version 2.4.2, GPL 3.0 ©2017, Darktable team) to adjust lighting. We calculated the diffuse light index (DLI) using the software Hemisfer (https://www.wsl.ch/de/services‐und‐produkte/software‐websites‐und‐apps/hemisfer.html, Schleppi et al., 2007).

### 2.5 Measures of stand structural complexity

Four different measures were used in this study to quantify stand structural complexity. Based on a full tree inventory of each plot, we calculated the total basal area (BA), representing stand density for all trees with a diameter at breast height (DBH) >7 cm (Storch et al., 2020).

Second, we calculated the coefficient of variation (CV) of DBH (DBH_cv) for each plot.

Third, to directly measure the geometric complexity of the distribution of plant material within the stand, we used data from terrestrial laser scans (TLS) to compute the index of stand structural complexity (SSCI) following the approach suggested by Ehbrecht et al. (2017). To obtain the geometric data, on each plot center, a single TLS was conducted between September 2017 and May 2018. Each scan was carried out with a Faro Focus 3D 120 (Faro Technologies Inc.) laser scanner set to 0.044° angular resolution. A full 360° horizontal and 150° vertical angular range was covered, resulting in a maximum of 29 million points per scan. The scanner was placed on a tripod at 1.3 m above the ground (Frey et al., 2019).

Finally, we used available aerial images with 20 cm pixel resolution and 60% forward and 30% side overlaps (State Office for Geoinformation and Rural Development, https://www.lgl‐bw.de/unsere‐themen/Produkte/Geodaten/Digitale‐Orthophotos/) to generate a digital surface model (DSM), using a structure from motion workflow (equivalent to Zielewska‐Büttner et al., 2016). We used Agisoft Photoscan commercial software for this purpose (v. 1.3.4, AgiSoft, St. Petersburg, 2017). From the resulting DSM with a resolution of 40 cm, we computed the Terrain Ruggedness Index (TRI, Wilson et al., 2007), as a measure of the geometric complexity of the crown surface (Frey et al., 2019).

### 2.6 Measures of resource heterogeneity

To analyze resource heterogeneity within each plot, we calculated the coefficient of variation (CV) of measured resource variables (light, soil chemistry), based on data from each of the six subplots. To assess potential intercorrelations among resource heterogeneity variables, we inspected both, a correlation matrix using the R‐package “corrplot” (Wei & Simko, 2017, Figure S1), and the first two axes of a principal component analysis using the “prcomp” function of the R‐package “stats” (version 3.6.3, R Core Team, 2020, Figure S1). These assessments showed that many soil variables were correlated with each other and could thus not be incorporated as independent predictors into our models. Furthermore, overfitting the models with many soil variables would have hampered the ecological interpretability of model outcomes, which we wanted to avoid (e.g., Cox, 2007). We thus selected soil pH and C:N ratio to reflect varying conditions of soil resources. Soil pH mostly correlated with micronutrients such as Mg and Ca, nitrate and negatively with Fe. C:N ratio correlated with ammonia, K and Na concentrations (more information about the variable selection process see Appendix Sec 1).

### 2.7 Classification of forest communities

The 135 plots contained a total of 332 vascular plant species, with species richness values ranging from 1 to 58. We classified the different forest communities according to their plant species composition. We log transformed and scaled the data of plant species occurrences at the 1‐ha plot level via the Hellinger method prior to data analysis (Legendre & Gallagher, 2001). We calculated dissimilarities in species composition using the Bray–Curtis distance and created hierarchical cluster dendrograms using option “ward.D2” for Ward clustering within the R package “vegan” (Murtagh & Legendre, 2014). To find the optimal numbers of clusters, we used the clustering method of the R package NbClust (Charrad et al., 2014); we chose “NULL” for distance, “ward.D2” as method, and “kl” as index.

This resulted in four clusters of forest plant communities (Figure S3) with the strongest differentiation in terms of species composition, due to environmental differences, such as soil chemistry, and altitude (Tables S1, S2, Figures S4 and S5). Moreover, we determined the phytosociological unit of each of these forest communities, using the indicator species analysis of the function “multipatt” from the R‐package “indicspecies” (Cáceres et al., 2010). This method calculates the extent to which certain species characterize the separation of one community from another. We then identified the communities according to Schubert et al. (2009) (Table S1) as Galio‐Abietetum, Vaccinio‐ and Luzulo‐Abietetum (Vac + Luz‐Abietetum), Galio‐ and Luzulo‐Fagetum (Gal+Luz‐Fagetum) and Pyrolo‐Abietetum.

### 2.8 Statistical analyses

We performed all statistical analyses in R version 3.6.3 (R Core Team, 2020). To fit our data to the most appropriate statistical distributions, we used the fitdistplus‐package (version 1.0–14, Delignette‐Muller & Dutang, 2015) for each response variable. Many potential explanatory variables were confounded in our study. We thus inspected intercorrelations using correlation matrices and principal component analyses (Figures S1 and S2). We finally defined CV of pH and C:N ratio concentrations of each plot as two variables that resembled soil resource heterogeneity.

For hypothesis H1, we tested whether the stand structural complexity measures (BA, DBH_cv, SSCI, and TRI) have an effect on any of the resource heterogeneity variables (DLI_cv, pH_cv, and C:N‐ratio_cv). For DLI_cv data, we assumed a normal distribution while the other two response variables were log‐normally distributed. We fitted generalized linear models (GLM) for each resource heterogeneity variable as separate responses and assessed each forest structural complexity variable as a predictor separately. Soil heterogeneity variables showed significant differences for the different forest communities (Table S2). We thus used the same fixed effects structure as described above, but included the forest community as a random effect in linear mixed effects models. These models were fitted using maximum likelihood (glmer function of the R‐package lme4 version 1.1–26, Bates et al., 2015). For the model selection with light heterogeneity as response, we did not include any soil variables in the models. Light heterogeneity is only indirectly influenced by soil conditions, affecting different plant abundances, which may be confounded with structural complexity variables in our study. We did not model such indirect pathways. The soil variables, however, may be influenced by light, due to increased decomposition of organic matter which is induced by increased microbiotic activity due to higher temperatures in mesic environments (Binkley, 1984; Covington, 1981). We performed model selection for models of each heterogeneity–structural complexity combination, using the “dredge” function of the MuMin‐package (Bartoń, 2019). The best candidate model was indicated by the lowest AIC value. To test whether mean values of the corresponding heterogeneity variable were responsible for the correlation with the stand structural complexity variable, they had to remain present in the best candidate model.

Next, (H2) we tested whether understory plant species richness was affected by the resource heterogeneity variables (DLI_cv, pH_cv and C:N‐ratio_cv). We fitted generalized linear mixed effect models, assuming a negative binomial distribution for species richness data and following the modeling approach outlined for H1. We used the glmer.nb function (R‐package MASS, version 7.3–51.5, Venables & Ripley, 2002), which internally calls “lme4” package to compute mixed effects. Additionally, we included absolute values of DLI, soil pH, and C:N ratio and all of their possible interactions with resource heterogeneity variables as covariates, to account for confounding effects between absolute resource availability and heterogeneity. As random factor, we selected the forest communities to account for initial species richness differences and performed a model selection using the “dredge” function of the MuMin‐package (Bartoń, 2019). The model with lowest AIC value was selected as best candidate model, with the requirement of including the mean values of the corresponding heterogeneity variables as additive fixed effects. Full models and best model candidates are presented in Table 1.

For each model, we calculated r‐squared values (Zhang, 2017). We used “r.squaredGLMM”‐function of the MuMin package and provided both marginal and conditional R² for linear mixed effects models (Nakagawa & Schielzeth, 2013). All final models were assessed in terms of outliers, normally and homogenously distributed residuals, using diagnostic plots. These assessments indicated no violations of model assumptions.

**TABLE 1:** Table of all performed model selections to test hypotheses

Hypo‐thesis	Full model (response and fixed effects)	Best candidate model (response and fixed effects)	Random effect	Distribution	*p*‐Value	*R* ^2^	conditional *R* ^2^
H1	DLI_cv ~ BA * DLI	DLI_cv ~ BA + DLI	–	Normal	<.001	.26	
H1	DLI_cv ~ DBHcv * DLI	DLI_cv ~ DBHcv + DLI	–	Normal	<.001	.32	
H1	DLI_cv ~ SSCI * DLI	DLI_cv ~ SSCI + DLI	–	Normal	<.001	.25	
H1	DLI_cv ~ TRI * DLI	DLI_cv ~ TRI + DLI	–	Normal	<.001	.16	
H1	pH_cv ~ BA * pH * DLI * DLI_cv	pH_cv ~ BA + DLI_cv + pH + BA:DLI_cv + BA:pH	(1|f. community)	Log‐normal	n.s.		
H1	pH_cv ~ DBHcv * pH * DLI * DLI_cv	pH_cv ~ DBHcv + DLI + DLI_cv + pH + DBHcv:DLI	(1|f. community)	Log‐normal	<.001	.003	.10
H1	pH_cv ~ SSCI * pH * DLI * DLI_cv	pH_cv ~ SSCI + DLI + pH + SSCI:pH	(1|f. community)	Log‐normal	n.s.		
H1	pH_cv ~ TRI * pH * DLI * DLI_cv	pH_cv ~ TRI + DLI + pH + TRI:DLI	(1|f. community)	Log‐normal	<.001	.11	.11
H1	CN‐ratio_cv ~ BA * CN‐ratio * DLI * DLI_cv	CN‐ratio_cv ~ BA + DLI	(1|f. community)	Log‐normal	n.s.		
H1	CN‐ratio_cv ~ DBHcv * CN‐ratio * DLI * DLI_cv	CN‐ratio_cv ~ CN‐ratio + DBHcv	(1|f. community)	Log‐normal	n.s.		
H1	CN‐ratio_cv ~ SSCI * CN‐ratio * DLI * DLI_cv	CN‐ratio_cv ~ CN‐ratio + DLI + DLI_cv + SSCI + CN‐ratio:DLI + CN‐ratio:SSCI + DLI_cv:SSCI + DLI:SSCI	(1|f. community)	Log‐normal	.008	.002	.002
H1	CN‐ratio_cv ~ TRI * CN‐ratio * DLI * DLI_cv	CN‐ratio_cv ~ CN‐ratio + DLI_cv + TRI + CN‐ratio:DLI_cv + CN‐ratio:TRI + DLI_cv:TRI + CN‐ratio:DLI_cv:TRI	(1|f. community)	Log‐normal	.005	.002	.002
H2	SR_herb_agg ~ DLI_cv * DLI * pH * pH_cv	SR_herb_agg ~ DLI + DLI_cv	(1|f. community)	Negative binomial	<.01	.03	.60
H2	SR_herb_agg ~ DLI_cv * DLI * CN‐ratio * CN‐ratio_cv	SR_herb_agg ~ CN‐ratio + CN‐ratio_cv + DLI + DLI_cv + CN‐ratio_cv:DLI_cv	(1|f. community)	Negative binomial	<.013	.07	.61

Note

“Full model” shows the initial model structure, with all possible interactions of fixed factors. The column “Best candidate model” shows the most parsimonious model structure, based on AIC. The column “Random effect” shows the random effect structure for mixed effects models. The “*p*‐value” represents the significance of the target predictor variable and its interaction if applicable. “Distribution” indicates the empirical probability distribution of data. The column “*R* ^2”^ reports marginal *R* ^2^ for mixed effects models. For these models, conditional R^2^ are given in addition.

## 3 RESULTS

### 3.1 Effects of structural complexity on resource heterogeneity (Hypothesis 1)

**FIGURE 3:**
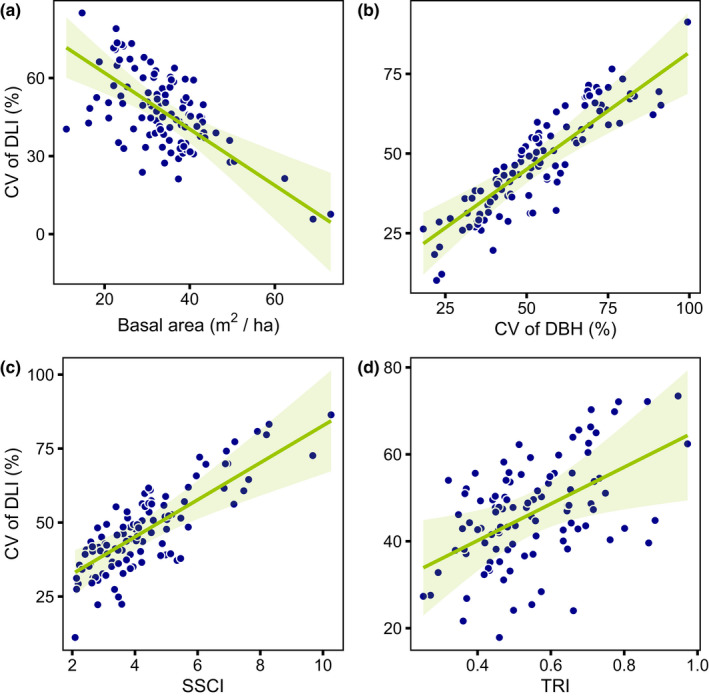
Hypothesis 1. Significant linear models (*p* < .05) between the forest structural complexity variables and the heterogeneity of light. The points show the predicted response values by the model of best fit. The regression line shows the prediction of the respective response where covariates are held constant at their respective means. The light green ribbons show the 95% confidence intervals

**FIGURE 4:**
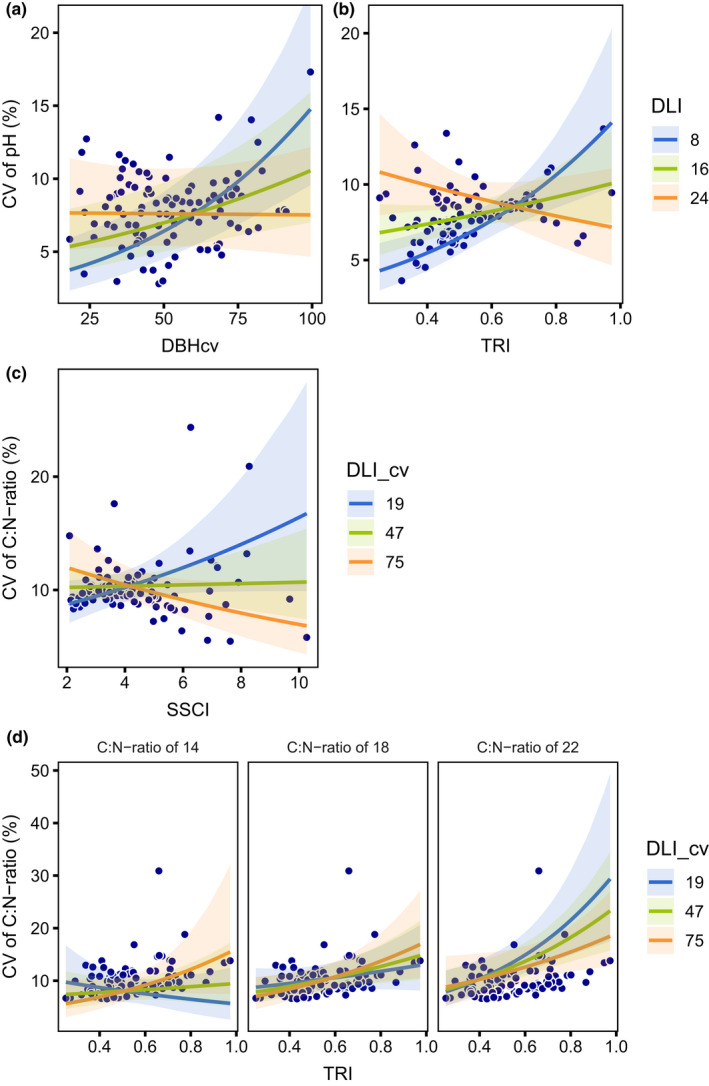
Hypothesis 1. Significant generalized linear mixed effects models (*p* < .05) between the forest structural complexity variables and the heterogeneity resources of soil pH and C:N ratio. The points show the predicted response values by the model of best fit. The regression lines show the marginal means of (a, b) pH–heterogeneity at different diffuse light indices levels (%), and (c, d) C:N ratio heterogeneity at different light heterogeneity levels, considering the significant interactions. The other covariates are held constant at their respective means. The transparent ribbons show the 95% confidence intervals

Forest structural complexity influenced heterogeneity of diffuse light index (Figure 3; Table 1). It also influenced the soil pH under low light intensity and C:N ratio when the coefficient of variation of DLI (DLI_cv) is low; for TRI, this effect was significant under lower DLI_cv, and under higher C:N ratio conditions (Figure 4; Table 1).

DLI_cv was negatively correlated with BA (Figure 3a, *t* = −4.51, p(BA) < 0.001, *r*² = .26) and positively correlated with DBH_cv (Figure 3b, *t* = 5.66, p(DBH) < 0.001, *r*² = .32). DLI_cv was also positively correlated with SSCI (Figure 3c, *t* = 4.45, p(SSCI) < 0.001, *r*² = .25), and with TRI (Figure 3d, *t* = 2.50, p(TRI) = 0.014, *r*² = .16).

The heterogeneity of soil pH (pH_cv) increased with DBH_cv at low light intensity, but not at high light intensity (Figure 4a, *t* (*df* = 107) = −4.11, p(DBH_cv:DLI interaction) <0.001, *r*² = .003). Similarly, soil pH_cv increased with TRI under low light intensities, but even decreased with TRI at high light intensities (Figure 4b, *t* (*df* = 101) = −3.50, p(TRI:DLI) < 0.001, *r*² = .25). The coefficient of variation of soil C:N ratio (C:N‐ratio_cv) increased with SSCI at low light heterogeneity levels and decreased at high levels (Figure 4c, *t* (*df* = 107) = −2.64, p(DLI cv:SSCI) = 0.008, *r*² = .002). Also, C:N‐ratio_cv increased with higher TRI at either low DLI_cv or high C:N ratio levels. (Figure 4d, *t* (107) = −2.78, p(CNratio:DLI cv:TRI) = 0.005, all model outputs in Tables S3–S10). Other forest complexity indices had no significant effect on any of the heterogeneity variables.

### 3.2 Effects of resource heterogeneity on plant species richness (Hypothesis 2)

Understory plant species richness was significantly affected by light heterogeneity, depending on the heterogeneity of soil C:N ratios.

**FIGURE 5:**
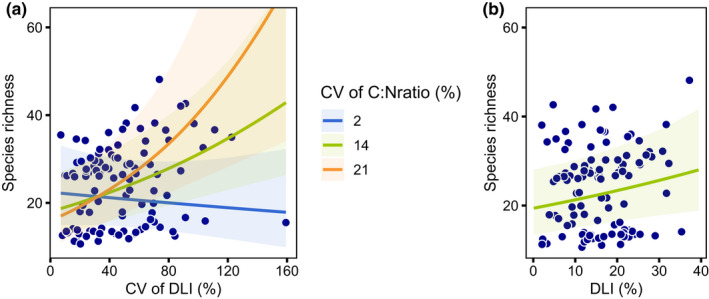
Hypothesis 2. The influence of light heterogeneity (a) and light intensity (b) on understory species richness. The points show the predicted response values by the model of best fit. The regression lines in (a) show marginal means of light heterogeneity at different C:N ratio levels, considering the significant interaction of these both variables (*p* = .02). Covariates are held constant at their mean. In (b) the regression shows the relation of species richness to light intensity in the same model (*p* = .03). The transparent ribbons show the respective 95% confidence intervals

The model showed that DLI_cv had a positive effect on plant species richness if C:N ratio_cv was also high (Figure 5a, *z* (*df* = 107) = 2.38, p(DLI_cv:C:N‐ratio_cv interaction) = 0.02, *r*² = 0.62). Understory species richness generally increased with increasing DLI (p(DLI) = 0.03, Figure 5b; model output in Table S11 and S12). Other heterogeneity variables had no significant effect on species richness.

## 4 DISCUSSION

### 4.1 Structurally complex forests create heterogeneity in resource availability (Hypothesis 1)

Our analyses indicated that forest structural complexity promotes the heterogeneity of light, soil pH, and C:N ratio at the forest floor.

Light heterogeneity was found to decrease with higher stand density (BA). Also, previous studies reported increasing light pre‐emption at the forest floor with increasing BA (Reich et al., 2012). However, our data showed that patterns of light heterogeneity (DLI_cv) were independent from light intensity (DLI) in our forest ecosystem (Figure S2). Stands with a very high BA also showed low values of other structural complexity variables (DBH_cv, SSCI, TRI; Table S2) and thus represent even‐aged forest stands dominated by spruce or beech trees. We found positive relationships between stand structural complexity and light heterogeneity.

By design, components of forest structural complexity are known to be influenced by forest management practices in our study system. Here, continuous‐cover forestry practices with selective tree harvesting and uneven‐aged tree structures represent stands with especially high structural complexity (Storch et al., 2020): DBH is usually positively related to tree age (Rohner et al., 2013) and a high coefficient of variation in this parameter may represent a broad age class distribution of trees. A decrease in crown density with very high tree age, for instance, leads to enhanced light transmission below the canopy (Nock et al., 2008). Study sites that were characterized by a high variability in trees of different ages can thus harbor various light conditions at the forest floor.

Besides DBH_cv, forest structural complexity is also represented by two other indices, SSCI and TRI. As opposed to DBH_cv, high values of the SSCI index, for instance, are associated with a higher geometric complexity in the distribution of all plant material within stands (Ehbrecht et al., 2017). Our results thereby support previous findings that stands with a high diversity of tree diameters coincide with stands of high SSCI (Ehbrecht et al., 2017; Figure S2). We found similar relationships for the Terrain Ruggedness Index (TRI), a measure of the geometric complexity of the crown surface (Wilson et al., 2007). Thus, we may conclude that both optical approaches are useful proxies to represent overall complexity of forest structure in our system. Overall, our results show that structural components in our model forests can modify the light regime in the understory, leading to higher light heterogeneity in more structurally complex stands. This is in line with much of the literature from different forest systems where similar findings are promoted (see e.g., Kumar et al., 2018 for boreal forests, Matsuo et al., 2021 for tropical secondary forests, Valladaraes & Guzmán, 2006 for Mediterranean forests).

The heterogeneity of soil resources was also affected by measures of forest structural complexity, but these effects depended on the general light intensity for pH_cv and light heterogeneity for C:N‐ratio_cv. Specifically, the heterogeneity of soil pH increased with increasing DBH_cv and TRI, but only if DLI levels were not too high. Most other studies either assessed the effects of forest structure, or light intensity, on soil properties, and this is, to our knowledge, the first study to empirically show an interaction effect between forest structure and light intensity. It is well known that increased nutrient release can be traced back to higher decomposition rates, induced by increased light and thus higher temperatures (Binkley, 1984; Covington, 1981). However, different tree species can also have profound effects on soil chemistry in their direct neighborhood due to the quality and quantity of their litter and/or nutrient uptake (e.g. Prescott, 2002; Rothe & Binkley, 2001). For example, pH can be spatially variable if different tree species have litter with various levels of calcium concentration, directly affecting soil acidity (Reich et al., 2005). In combination, it is currently under debate whether and how structural vegetation properties modulate the effects of light‐induced soil properties (Badraghi et al., 2021). Laboratory incubation studies have shown that processes affecting soil nutrient status are influenced by an interaction between litter quality and temperature (Fierer et al., 2005). Decomposition rates of low‐quality litter material are thereby more temperature sensitive than the decomposition of high‐quality litter (Fierer et al., 2005). However, in our large‐scale observational study, we assume that the interaction patterns we found could also be related to our experimental design. The negative correlation between DLI and DBH_cv (Figure S2) indicates that structurally simple stands sometimes coincided with conditions of high light intensity. Such stands were often former even‐aged spruce or beech forests, which were only recently managed with a single‐tree harvest. Subplots in our study may have coincided with large, open gaps that were characterized by a homogeneous coverage of brambles (*Rubus fruticosus* agg.), potentially inducing the shedding of a homogeneous litter material across the plot. For plots that did not follow these characteristics, the positive relationship between structural complexity and heterogeneity on soil pH_cv was presumably due to increasing heterogeneity of litter of different quality and quantity material across the plot (Reich et al., 2005).

The interaction effects on soil C:N‐ratio_cv may be specific to our design. We only found positive correlations between stand structural complexity (represented by either SSCI or TRI), when DLI_cv was low, or *vice versa*. As outlined above, both stand structural complexity variables were positively correlated with DLI_cv, and this confounding may have rendered one variable less powerful when both are included in our model.

Although these interaction effects may be partly related to design in our case, the discussion calls for future research on the relative importance of plant litter quality and light intensity in mountainous temperate forest soils (Badraghi et al., 2021), where both effects should be experimentally separated to improve our understanding on that matter.

### 4.2 Resource heterogeneity increases plant diversity (Hypothesis 2)

The heterogeneity–diversity relationship is one of the central hypotheses explaining the diversity of plant species (Chesson, 2000; Stein et al., 2014). Our findings generally support this view. However, we found independent effects of light intensity (DLI) and resource heterogeneity (soil and light resources) on species richness in our study system.

The relative importance of resource quantity and heterogeneity in driving understory plant species richness has been previously reviewed (Bartels & Chen, 2010; Su et al., 2019). In general terms, both aspects are important drivers of understory plant species richness in forests (Bartels & Chen, 2010; Reich et al., 2012; Su et al., 2019). It can be generalized that resource availability, especially in terms of light, is an important promoter of understory plant species richness in young and mature forest stands with little disturbance (Bartels & Chen, 2010). Light availability also plays an important role in stands that are dominated by single tree species (Su et al., 2019). Yet resource heterogeneity gains an important role as a driver of understory plant species richness in disturbed stands, or at later successional stages that are characterized by a mixed overstory (Bartels & Chen, 2010; Su et al., 2019). Our mountainous temporal forest system may represent structures in which both factors, resource availability and heterogeneity, are important.

Similar to previous studies, we found a positive relationship between light availability and understory plant species richness (Dormann et al., 2020; Reich et al., 2012). In European temperate forests, this supports the view that plants below the tree canopy are generally limited in light availability (Dormann et al., 2020). However, Dormann et al. (2020) found no significant improvement by adding light heterogeneity as a predictor of plant species richness in their models. Theoretically, an increase in structural complexity can cause an increase in the spatio‐temporal diversity of moving sun flecks at the forest floor. The proportion of sun flecks in the understory of a forest is highly correlated with the proportion of diffuse light penetrating the canopy (Parent & Messier, 1996). In these sun flecks, the amount of light can be over two orders of magnitude higher than in the shade (Chazdon & Pearcy, 1991). This can be beneficial for some species which are able to handle the sudden increase, or detrimental for those which are sensitive to full sunlight. Hence, on a very small scale, numerous species could be adapted to the many different light conditions present over space and time. Our data may add an important explanation for why light heterogeneity is still not found to be a strong predictor of plant species richness in some temperate forests (Dormann et al., 2020): Light heterogeneity only affects species richness in combination with heterogeneous soil conditions. To the best of our knowledge, we are the first one to report such interactions.

The ratio of soil C:N content represents the type of humus at forest floor (Leuschner et al., 2017), which may potentially offer different soil‐related niche spaces for understory plant species. Harpole and Tilman (2007) proposed the “niche dimension hypothesis” in which species numbers are determined by the number of limiting resources (Harpole et al., 2017). This hypothesis was corroborated by empirical data, showing that variable nutrient ratios within soils enhance niche space and provide conditions for coexistence of many species (Harpole et al., 2016). At the 1‐ha plot level in our study, the heterogeneity of C:N ratios could represent one aspect of niche dimensionality. Under low‐resource heterogeneity, soil‐related niche dimension is small, which decreases the number of understory plant species that can successfully establish and coexist. Light is only seen as another dimension of niche space in this model (Harpole et al., 2017), and a large heterogeneity of light conditions at plot level would increase this dimension. Hence, it is not surprising to observe amplified effects of light heterogeneity on species richness, with higher heterogeneity of soil resources. In this context, it would be interesting to analyze study outcomes of heterogeneity–diversity relationship in the context of the niche space theory. Large resource heterogeneity at plot scale may thereby coincide with large niche space, which would imply that both theories: the heterogeneity–diversity hypothesis and the niche dimension hypothesis, provide sound explanation of species coexistence within communities.

## 5 CONCLUSION

This study supports the assumption that higher stand structural complexity promotes resource heterogeneity below canopies in mountainous temperate forest stands. For the heterogeneity of soil resources, we argue that interactions between general light conditions and litter material of different quality may be essential. Our findings also support the heterogeneity–diversity hypothesis in our system. However, as opposed to previous studies, we showed that light heterogeneity is an important driver of understory richness, in combination with a high soil resource heterogeneity. This interaction might be explained by niche–dimension theory and could be a baseline for future studies that aim to understand below canopy species richness in temperate forest stands.

## CONFLICT OF INTEREST

The authors declare no conflict of interest.

## AUTHOR CONTRIBUTION

**Jan Helbach:** Conceptualization (equal); Data curation (lead); Formal analysis (lead); Investigation (lead); Methodology (equal); Visualization (lead); Writing – original draft (lead); Writing – review & editing (equal). **Julian Frey:** Data curation (supporting); Investigation (supporting); Writing – review & editing (supporting). **Christian Messier:** Writing – review & editing (equal). **Martin Mörsdorf:** Formal analysis (equal); Supervision (supporting); Validation (equal); Writing – review & editing (equal). **Michael Scherer‐Lorenzen:** Conceptualization (equal); Funding acquisition (lead); Methodology (equal); Project administration (lead); Validation (equal); Writing – original draft (equal); Writing – review & editing (equal).

## Supporting information

Supplementary Material

## Data Availability

All data used to perform the analyses associated with this study have been archived on Dryad (https://doi.org/10.5061/dryad.kwh70rz52).
