# Supplementary material for: Light heterogeneity affects understory plant species richness in temperate forests supporting the heterogeneity–diversity hypothesis
Source: Ecol Evol. 2022 Feb 19;12(2):e8534. doi: 10.1002/ece3.8534 (PMC8858222; doi:10.1002/ece3.8534)
Supplement: Supplementary file 1 — Supplementary Material [file ECE3-12-e8534-s001.docx]

Supporting Information

# Light heterogeneity affects understory plant species richness in temperate forests supporting the heterogeneity-diversity hypothesis

**Jan Helbach¹, Julian Frey^2,4^, Christian Messier^3^ Martin Mörsdorf¹ & Michael Scherer-Lorenzen¹**

1. Geobotany, Faculty of Biology, University of Freiburg, Schänzlestr. 1, 79104 Freiburg, Germany

2. Chair of Remote Sensing and Landscape Information Systems, Faculty of Environment and Natural Resources, University of Freiburg, Tennenbacherstr. 4, 79106 Freiburg, Germany

3. CEF, ISFORT, Université du Québec en Outaouais et à Montréal, Montréal, Canada

4. Current address: Chair of Forest Growth, Faculty of Environment and Natural Resources, University of Freiburg, Tennenbacherstr. 4, 79106 Freiburg, Germany

Correspondance: Michael Scherer-Lorenzen (michael.scherer@biologie.uni-freiburg.de), University of Freiburg, Schänzlestraße 1, 79104 Freiburg, Germany

# Variable selection

In our study we analyzed many factors. To justify which variable was put into which model we did a comprehensive variable selection. In the first step we investigated the correlation of soil variables, as it is very well known that many variables in the soil are auto–correlated. The Correlation matrix (Fig. S1, left) show that C:N-ratio, K, Ammonia and Na, are highly correlated. For the analysis we used C:N-ratio because it includes already two of the variables and the measure was more precise (see methods).


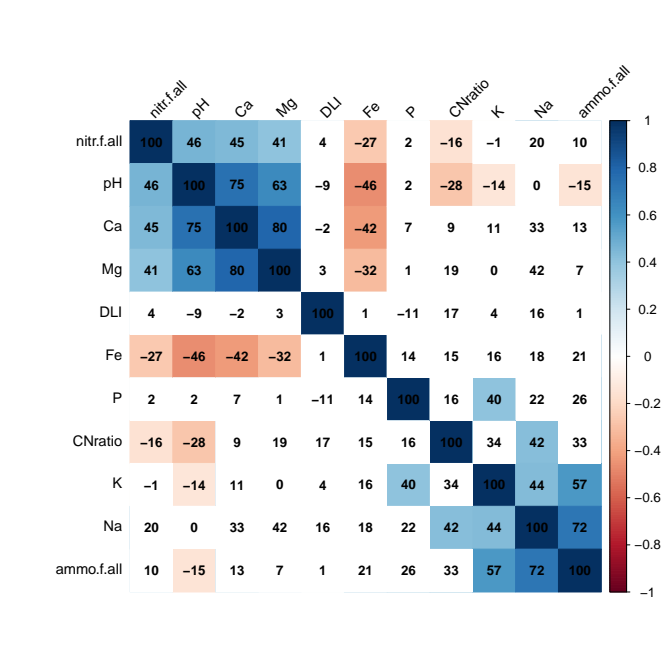

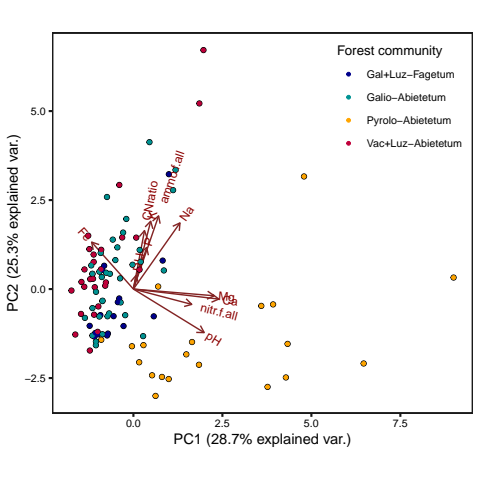


Figure S1: Auto–correlation analyses of the soil factors. Left: Correlation matrix, values indicate correlation in %. Colors indicate the strength of the correlation. When the correlation was significant ( p < 0.1) cells are colored. PCA of the analyzed factors (right). Arrows show the direction and the correlation with the axis. Points indicate the position of the plot, color indicates the affiliation of the forest community.

The second block of correlated variables are nitrate, pH, Ca, Mg and Fe negatively. Ca correlates most with the other variables, but is less easy to handle because of its high scatter of values. We thus decided to use pH as it was one of the variables which explained much of the variation of species richness. Also in the principle component analysis (PCA) mainly three soil variables are independent: pH on PC–axis 1 and C:N-ratio on PC–axis 2. P did not respond to the first two PC–axis and did not correlate with any of the other selected variables (Fig. S1, right). Thus nominated as third soil variable for Hypothesis 1.

In Fig. S2 we show the correlation of the variables selected in the two hypothesis. As the forest communities are binomial variables, they cannot be processed within a correlation matrix or a PCA. However, in the letter we can mark the plots according to the forest communities. For H1 we can exclude altitude which was a proxy for temperature and season length. However it is highly negatively correlated with other variables such as pH, P_cv, DLI_cv and others. The other correlations are incorporated in the modeling process under ecological aspects.


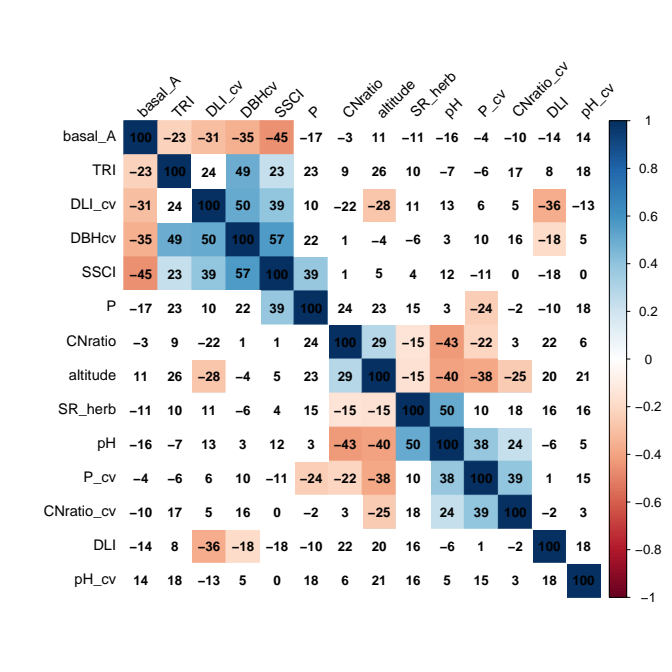

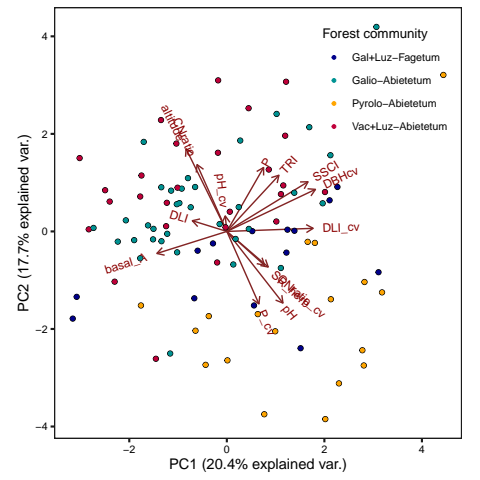


Figure S2: In these graphs we demonstrate the relationship of our predictors and responses for the two hypothesis. Left: Correlation matrix, values indicate correlation in %. Colors indicate the strength of the correlation. When the correlation was significant ( p < 0.1) cells are colored. PCA of the analyzed factors (right). Arrows show the direction and the correlation with the axis. Points indicate the position of the plot, color indicates the affiliation of the forest community.

# Classification of forest communities

The plots of our investigation area vary in there species composition. Thus, we classified our plots into different forest communities according to their species composition (Fig. S3 & S4).

Table S1: Output of the indicator species classification. For each species, the combination with a highest association value was chosen. Best matching patterns are tested for statistical significance of the forest communities (p-value). Further information in main manuscript.

| Species | Gruppenindex | Propability for determination | p-value |
| --- | --- | --- | --- |
| Senecio ovatus | Galio-Abietetum | 0.66 | 0.00 |
| Veronica officinalis | Galio-Abietetum | 0.61 | 0.00 |
| Sambucus racemosa | Galio-Abietetum | 0.60 | 0.00 |
| Rubus idaeus | Galio-Abietetum | 0.60 | 0.00 |
| Oxalis acetosella | Galio-Abietetum | 0.59 | 0.00 |
| Mycelis muralis | Galio-Abietetum | 0.59 | 0.00 |
| Dryopteris dilatata | Galio-Abietetum | 0.58 | 0.00 |
| Athyrium filix femina | Galio-Abietetum | 0.58 | 0.00 |
| Prenanthes purpurea | Galio-Abietetum | 0.57 | 0.00 |
| Moehringia trinervia | Galio-Abietetum | 0.53 | 0.00 |
| Agrostis capillaris | Galio-Abietetum | 0.52 | 0.01 |
| Carex pilulifera | Galio-Abietetum | 0.52 | 0.01 |
| Galium rotundifolium | Galio-Abietetum | 0.51 | 0.00 |
| Impatiens noli-tangere | Galio-Abietetum | 0.51 | 0.00 |
| Ajuga reptans | Galio-Abietetum | 0.49 | 0.01 |
| Epilobium montanum | Galio-Abietetum | 0.47 | 0.03 |
| Circaea alpina | Galio-Abietetum | 0.42 | 0.01 |
| Agrostis stolonifera | Galio-Abietetum | 0.42 | 0.00 |
| Digitalis purpurea | Galio-Abietetum | 0.41 | 0.01 |
| Calamagrostis arundinacea | Galio-Abietetum | 0.40 | 0.00 |
| Stellaria nemorum | Galio-Abietetum | 0.37 | 0.02 |
| Epilobium angustifolium | Galio-Abietetum | 0.37 | 0.05 |
| Phegopteris connectilis | Galio-Abietetum | 0.36 | 0.01 |
| Senecio hercynicus | Galio-Abietetum | 0.35 | 0.02 |
| Paris quadrifolia | Galio-Abietetum | 0.34 | 0.06 |
| Melampyrum pratense | Galio-Abietetum | 0.30 | 0.11 |
| Adenostyles alliariae | Galio-Abietetum | 0.29 | 0.05 |
| Hypochaeris radicata | Galio-Abietetum | 0.29 | 0.05 |
| Gymnocarpium dryopteris | Galio-Abietetum | 0.28 | 0.13 |
| Blechnum spicant | Galio-Abietetum | 0.28 | 0.21 |
| Holcus mollis | Galio-Abietetum | 0.26 | 0.11 |
| Hieracium lachenalii | Galio-Abietetum | 0.26 | 0.10 |
| Carex muricata agg. | Galio-Abietetum | 0.24 | 0.34 |
| Silene dioica | Galio-Abietetum | 0.24 | 0.16 |
| Solidago virgaurea | Galio-Abietetum | 0.23 | 0.63 |
| Scrophularia nodosa | Galio-Abietetum | 0.23 | 0.47 |
| Linaria vulgaris | Galio-Abietetum | 0.21 | 0.27 |
| Lysimachia nemorum | Galio-Abietetum | 0.21 | 0.27 |
| Rumex acetosella | Galio-Abietetum | 0.21 | 0.27 |
| Veronica montana | Galio-Abietetum | 0.21 | 0.27 |
| Galium saxatile | Galio-Abietetum | 0.20 | 0.60 |
| Dryopteris carthusiana | Galio-Abietetum | 0.20 | 0.66 |
| Huperzia selago | Galio-Abietetum | 0.18 | 0.56 |
| Cytisus scoparius | Galio-Abietetum | 0.17 | 0.79 |
| Epilobium obscurum | Galio-Abietetum | 0.17 | 0.49 |
| Sonchus oleraceus | Galio-Abietetum | 0.16 | 0.56 |
| Carex pairae | Galio-Abietetum | 0.15 | 1.00 |
| Carex pallescens | Galio-Abietetum | 0.15 | 1.00 |
| Carex rostrata | Galio-Abietetum | 0.15 | 1.00 |
| Cephalanthera damasonium | Galio-Abietetum | 0.15 | 1.00 |
| Chaerophyllum aureum | Galio-Abietetum | 0.15 | 1.00 |
| Circaea x intermedia | Galio-Abietetum | 0.15 | 1.00 |
| Dactylis polygama | Galio-Abietetum | 0.15 | 1.00 |
| Dactylorhiza maculata | Galio-Abietetum | 0.15 | 1.00 |
| Digitalis lutea | Galio-Abietetum | 0.15 | 1.00 |
| Epilobium hirsutum | Galio-Abietetum | 0.15 | 1.00 |
| Genista sagittalis | Galio-Abietetum | 0.15 | 1.00 |
| Hieracium laevigatum | Galio-Abietetum | 0.15 | 1.00 |
| Leontodon helveticus | Galio-Abietetum | 0.15 | 1.00 |
| Plantago lanceolata | Galio-Abietetum | 0.15 | 1.00 |
| Potentilla erecta | Galio-Abietetum | 0.15 | 1.00 |
| Rumex acetosa | Galio-Abietetum | 0.15 | 1.00 |
| Rumex arifolius | Galio-Abietetum | 0.15 | 1.00 |
| Stellaria aquatica | Galio-Abietetum | 0.15 | 1.00 |
| Thuja sp. | Galio-Abietetum | 0.15 | 1.00 |
| Crepis paludosa | Galio-Abietetum | 0.12 | 0.95 |
| Lycopodium annotinum | Galio-Abietetum | 0.12 | 1.00 |
| Vaccinium myrtillus | Vac+Luz-Abietetum | 0.81 | 0.00 |
| Deschampsia flexuosa | Vac+Luz-Abietetum | 0.68 | 0.00 |
| Picea abies | Vac+Luz-Abietetum | 0.66 | 0.00 |
| Vaccinium vitis-idaea | Vac+Luz-Abietetum | 0.62 | 0.00 |
| Sorbus aucuparia | Vac+Luz-Abietetum | 0.55 | 0.00 |
| Abies alba | Vac+Luz-Abietetum | 0.50 | 0.25 |
| Calluna vulgaris | Vac+Luz-Abietetum | 0.33 | 0.03 |
| Pinus sylvestris | Vac+Luz-Abietetum | 0.27 | 0.30 |
| Melampyrum sylvaticum | Vac+Luz-Abietetum | 0.26 | 0.34 |
| Pteridium aquilinum | Vac+Luz-Abietetum | 0.23 | 0.30 |
| Carex echinata | Vac+Luz-Abietetum | 0.20 | 0.28 |
| Betula pendula | Vac+Luz-Abietetum | 0.20 | 0.27 |
| Eriophorum vaginatum | Vac+Luz-Abietetum | 0.16 | 0.69 |
| Molinia caerulea | Vac+Luz-Abietetum | 0.16 | 0.69 |
| Pinus rotundata | Vac+Luz-Abietetum | 0.16 | 0.69 |
| Vaccinium oxycoccos | Vac+Luz-Abietetum | 0.16 | 0.69 |
| Vaccinium uliginosum | Vac+Luz-Abietetum | 0.16 | 0.69 |
| Galium uliginosum | Vac+Luz-Abietetum | 0.13 | 0.67 |
| Hieracium pilosella | Vac+Luz-Abietetum | 0.13 | 0.64 |
| Rubus fruticosus agg. | Gal+Luz-Fagetum | 0.70 | 0.00 |
| Teucrium scorodonia | Gal+Luz-Fagetum | 0.64 | 0.00 |
| Festuca altissima | Gal+Luz-Fagetum | 0.62 | 0.00 |
| Fagus sylvatica | Gal+Luz-Fagetum | 0.62 | 0.00 |
| Dryopteris filix-mas | Gal+Luz-Fagetum | 0.59 | 0.00 |
| Pseudotsuga menziesii | Gal+Luz-Fagetum | 0.50 | 0.00 |
| Luzula luzuloides | Gal+Luz-Fagetum | 0.49 | 0.01 |
| Impatiens glandulifera | Gal+Luz-Fagetum | 0.48 | 0.00 |
| Luzula sylvatica | Gal+Luz-Fagetum | 0.48 | 0.01 |
| Ilex aquifolium | Gal+Luz-Fagetum | 0.43 | 0.00 |
| Castanea sativa | Gal+Luz-Fagetum | 0.40 | 0.00 |
| Quercus petraea | Gal+Luz-Fagetum | 0.40 | 0.01 |
| Dryopteris affinis | Gal+Luz-Fagetum | 0.38 | 0.01 |
| Carex pendula | Gal+Luz-Fagetum | 0.35 | 0.01 |
| Impatiens parviflora | Gal+Luz-Fagetum | 0.35 | 0.01 |
| Carpinus betulus | Gal+Luz-Fagetum | 0.28 | 0.03 |
| Lonicera periclymenum | Gal+Luz-Fagetum | 0.28 | 0.03 |
| Sambucus sp. | Gal+Luz-Fagetum | 0.28 | 0.03 |
| Oreopteris limbosperma | Gal+Luz-Fagetum | 0.27 | 0.10 |
| Galeopsis tetrahit | Gal+Luz-Fagetum | 0.26 | 0.04 |
| Carex sp. | Gal+Luz-Fagetum | 0.25 | 0.15 |
| Quercus sp. | Gal+Luz-Fagetum | 0.25 | 0.14 |
| Eupatorium cannabinum | Gal+Luz-Fagetum | 0.25 | 0.28 |
| Hypericum perforatum | Gal+Luz-Fagetum | 0.24 | 0.22 |
| Melica uniflora | Gal+Luz-Fagetum | 0.24 | 0.15 |
| Juncus effusus | Gal+Luz-Fagetum | 0.24 | 0.49 |
| Cardamine flexuosa | Gal+Luz-Fagetum | 0.23 | 0.40 |
| Athyrium distentifolium | Gal+Luz-Fagetum | 0.22 | 0.28 |
| Salix caprea | Gal+Luz-Fagetum | 0.21 | 0.43 |
| Asplenium trichomanes | Gal+Luz-Fagetum | 0.20 | 0.19 |
| Carex brizoides | Gal+Luz-Fagetum | 0.20 | 0.18 |
| Holcus lanatus | Gal+Luz-Fagetum | 0.20 | 0.18 |
| Ulmus sp. | Gal+Luz-Fagetum | 0.20 | 0.17 |
| Poa nemoralis | Gal+Luz-Fagetum | 0.18 | 0.35 |
| Solanum dulcamara | Gal+Luz-Fagetum | 0.17 | 0.32 |
| Hypericum pulchrum | Gal+Luz-Fagetum | 0.16 | 0.60 |
| Hypericum humifusum | Gal+Luz-Fagetum | 0.15 | 0.58 |
| Betula pubescens | Gal+Luz-Fagetum | 0.11 | 0.90 |
| Brachypodium sylvaticum | Pyrolo-Abietetum | 0.84 | 0.00 |
| Carex sylvatica | Pyrolo-Abietetum | 0.77 | 0.00 |
| Lonicera xylosteum | Pyrolo-Abietetum | 0.76 | 0.00 |
| Fragaria vesca | Pyrolo-Abietetum | 0.76 | 0.00 |
| Carex flacca | Pyrolo-Abietetum | 0.75 | 0.00 |
| Viola reichenbachiana | Pyrolo-Abietetum | 0.75 | 0.00 |
| Galium odoratum | Pyrolo-Abietetum | 0.72 | 0.00 |
| Fraxinus excelsior | Pyrolo-Abietetum | 0.65 | 0.00 |
| Acer pseudoplatanus | Pyrolo-Abietetum | 0.60 | 0.00 |
| Hieracium murorum | Pyrolo-Abietetum | 0.60 | 0.00 |
| Corylus avellana | Pyrolo-Abietetum | 0.60 | 0.00 |
| Lathyrus vernus | Pyrolo-Abietetum | 0.58 | 0.00 |
| Mercurialis perennis | Pyrolo-Abietetum | 0.57 | 0.00 |
| Daphne mezereum | Pyrolo-Abietetum | 0.54 | 0.00 |
| Viburnum lantana | Pyrolo-Abietetum | 0.54 | 0.00 |
| Geranium robertianum | Pyrolo-Abietetum | 0.53 | 0.00 |
| Festuca gigantea | Pyrolo-Abietetum | 0.53 | 0.00 |
| Galeobdolon luteum agg. | Pyrolo-Abietetum | 0.53 | 0.00 |
| Convallaria majalis | Pyrolo-Abietetum | 0.51 | 0.00 |
| Crataegus monogyna | Pyrolo-Abietetum | 0.51 | 0.00 |
| Rosa sp. | Pyrolo-Abietetum | 0.51 | 0.00 |
| Prunus avium | Pyrolo-Abietetum | 0.50 | 0.00 |
| Sanicula europaea | Pyrolo-Abietetum | 0.49 | 0.00 |
| Vicia sepium | Pyrolo-Abietetum | 0.49 | 0.00 |
| Milium effusum | Pyrolo-Abietetum | 0.48 | 0.00 |
| Bromus ramosus agg. | Pyrolo-Abietetum | 0.47 | 0.00 |
| Cirsium arvense | Pyrolo-Abietetum | 0.47 | 0.00 |
| Primula elatior | Pyrolo-Abietetum | 0.47 | 0.00 |
| Sorbus aria | Pyrolo-Abietetum | 0.47 | 0.00 |
| Circaea lutetiana | Pyrolo-Abietetum | 0.46 | 0.00 |
| Carex digitata | Pyrolo-Abietetum | 0.46 | 0.00 |
| Angelica sylvestris | Pyrolo-Abietetum | 0.45 | 0.00 |
| Acer platanoides | Pyrolo-Abietetum | 0.45 | 0.00 |
| Hedera helix | Pyrolo-Abietetum | 0.45 | 0.00 |
| Epipactis helleborine | Pyrolo-Abietetum | 0.44 | 0.00 |
| Clematis vitalba | Pyrolo-Abietetum | 0.43 | 0.00 |
| Geum urbanum | Pyrolo-Abietetum | 0.43 | 0.00 |
| Hordelymus europaeus | Pyrolo-Abietetum | 0.43 | 0.00 |
| Lathyrus pratensis | Pyrolo-Abietetum | 0.43 | 0.00 |
| Ligustrum vulgare | Pyrolo-Abietetum | 0.43 | 0.00 |
| Sambucus nigra | Pyrolo-Abietetum | 0.43 | 0.01 |
| Lapsana communis | Pyrolo-Abietetum | 0.42 | 0.00 |
| Urtica dioica | Pyrolo-Abietetum | 0.42 | 0.03 |
| Stachys sylvatica | Pyrolo-Abietetum | 0.41 | 0.00 |
| Lonicera nigra | Pyrolo-Abietetum | 0.41 | 0.01 |
| Carex remota | Pyrolo-Abietetum | 0.40 | 0.01 |
| Cirsium oleraceum | Pyrolo-Abietetum | 0.38 | 0.00 |
| Prunus spinosa | Pyrolo-Abietetum | 0.38 | 0.00 |
| Ulmus glabra | Pyrolo-Abietetum | 0.38 | 0.00 |
| Galium mollugo | Pyrolo-Abietetum | 0.38 | 0.00 |
| Veronica chamaedrys | Pyrolo-Abietetum | 0.37 | 0.02 |
| Anemone nemorosa | Pyrolo-Abietetum | 0.36 | 0.01 |
| Maianthemum bifolium | Pyrolo-Abietetum | 0.34 | 0.05 |
| Poa chaixii | Pyrolo-Abietetum | 0.34 | 0.03 |
| Cornus sanguinea | Pyrolo-Abietetum | 0.33 | 0.02 |
| Dioscorea communis | Pyrolo-Abietetum | 0.33 | 0.01 |
| Euphorbia cyparissias | Pyrolo-Abietetum | 0.33 | 0.03 |
| Glechoma hederacea | Pyrolo-Abietetum | 0.33 | 0.02 |
| Orthilia secunda | Pyrolo-Abietetum | 0.33 | 0.01 |
| Prunella vulgaris | Pyrolo-Abietetum | 0.33 | 0.02 |
| Pulmonaria obscura | Pyrolo-Abietetum | 0.33 | 0.01 |
| Dactylis glomerata | Pyrolo-Abietetum | 0.32 | 0.02 |
| Equisetum sylvaticum | Pyrolo-Abietetum | 0.31 | 0.05 |
| Cirsium palustre | Pyrolo-Abietetum | 0.31 | 0.04 |
| Deschampsia cespitosa | Pyrolo-Abietetum | 0.31 | 0.07 |
| Ranunculus repens | Pyrolo-Abietetum | 0.30 | 0.03 |
| Aruncus dioicus | Pyrolo-Abietetum | 0.30 | 0.05 |
| Polygonatum verticillatum | Pyrolo-Abietetum | 0.30 | 0.12 |
| Melica nutans | Pyrolo-Abietetum | 0.29 | 0.05 |
| Viola hirta | Pyrolo-Abietetum | 0.29 | 0.03 |
| Polygonatum multiflorum | Pyrolo-Abietetum | 0.28 | 0.08 |
| Acer campestre | Pyrolo-Abietetum | 0.27 | 0.07 |
| Actaea spicata | Pyrolo-Abietetum | 0.27 | 0.07 |
| Alnus incana | Pyrolo-Abietetum | 0.27 | 0.06 |
| Brachypodium pinnatum | Pyrolo-Abietetum | 0.27 | 0.06 |
| Cephalanthera longifolia | Pyrolo-Abietetum | 0.27 | 0.07 |
| Euonymus europaea | Pyrolo-Abietetum | 0.27 | 0.08 |
| Juncus inflexus | Pyrolo-Abietetum | 0.27 | 0.08 |
| Leontodon hispidus | Pyrolo-Abietetum | 0.27 | 0.07 |
| Origanum vulgare | Pyrolo-Abietetum | 0.27 | 0.07 |
| Platanthera bifolia | Pyrolo-Abietetum | 0.27 | 0.07 |
| Primula veris | Pyrolo-Abietetum | 0.27 | 0.05 |
| Ribes rubrum | Pyrolo-Abietetum | 0.27 | 0.10 |
| Rubus caesius | Pyrolo-Abietetum | 0.27 | 0.06 |
| Sambucus ebulus | Pyrolo-Abietetum | 0.27 | 0.07 |
| Viburnum opulus | Pyrolo-Abietetum | 0.27 | 0.08 |
| Viola mirabilis | Pyrolo-Abietetum | 0.27 | 0.07 |
| Frangula alnus | Pyrolo-Abietetum | 0.26 | 0.46 |
| Hieracium sp. | Pyrolo-Abietetum | 0.24 | 0.12 |
| Alliaria petiolata | Pyrolo-Abietetum | 0.24 | 0.15 |
| Petasites albus | Pyrolo-Abietetum | 0.23 | 0.18 |
| Populus tremula | Pyrolo-Abietetum | 0.23 | 0.25 |
| Lysimachia nummularia | Pyrolo-Abietetum | 0.23 | 0.39 |
| Galium aparine | Pyrolo-Abietetum | 0.21 | 0.32 |
| Luzula pilosa | Pyrolo-Abietetum | 0.20 | 0.35 |
| Aegopodium podagraria | Pyrolo-Abietetum | 0.19 | 0.39 |
| Agrimonia eupatoria | Pyrolo-Abietetum | 0.19 | 0.39 |
| Aquilegia vulgaris | Pyrolo-Abietetum | 0.19 | 0.39 |
| Asarum europaeum | Pyrolo-Abietetum | 0.19 | 0.39 |
| Berberis vulgaris | Pyrolo-Abietetum | 0.19 | 0.39 |
| Calamintha menthifolia | Pyrolo-Abietetum | 0.19 | 0.39 |
| Carex alba | Pyrolo-Abietetum | 0.19 | 0.38 |
| Carex caryophyllea | Pyrolo-Abietetum | 0.19 | 0.37 |
| Carex nigra | Pyrolo-Abietetum | 0.19 | 0.37 |
| Carex ornithopoda | Pyrolo-Abietetum | 0.19 | 0.36 |
| Cerastium sp. | Pyrolo-Abietetum | 0.19 | 0.38 |
| Chrysosplenium alternifolium | Pyrolo-Abietetum | 0.19 | 0.38 |
| Cirsium vulgare | Pyrolo-Abietetum | 0.19 | 0.38 |
| Cornus mas | Pyrolo-Abietetum | 0.19 | 0.39 |
| Crataegus laevigata | Pyrolo-Abietetum | 0.19 | 0.38 |
| Elymus repens | Pyrolo-Abietetum | 0.19 | 0.39 |
| Epipactis atrorubens | Pyrolo-Abietetum | 0.19 | 0.36 |
| Epipactis purpurata | Pyrolo-Abietetum | 0.19 | 0.35 |
| Euphorbia verrucosa | Pyrolo-Abietetum | 0.19 | 0.39 |
| Filipendula ulmaria | Pyrolo-Abietetum | 0.19 | 0.38 |
| Galium palustre | Pyrolo-Abietetum | 0.19 | 0.38 |
| Hepatica nobilis | Pyrolo-Abietetum | 0.19 | 0.39 |
| Inula conyzae | Pyrolo-Abietetum | 0.19 | 0.39 |
| Juglans regia | Pyrolo-Abietetum | 0.19 | 0.34 |
| Lotus pedunculatus | Pyrolo-Abietetum | 0.19 | 0.38 |
| Mentha longifolia | Pyrolo-Abietetum | 0.19 | 0.39 |
| Petasites hybridus | Pyrolo-Abietetum | 0.19 | 0.38 |
| Potentilla reptans | Pyrolo-Abietetum | 0.19 | 0.39 |
| Reseda alba | Pyrolo-Abietetum | 0.19 | 0.38 |
| Robinia pseudoacacia | Pyrolo-Abietetum | 0.19 | 0.39 |
| Rubus saxatilis | Pyrolo-Abietetum | 0.19 | 0.39 |
| Rumex obtusifolius | Pyrolo-Abietetum | 0.19 | 0.39 |
| Salix cinerea | Pyrolo-Abietetum | 0.19 | 0.38 |
| Salvia glutinosa | Pyrolo-Abietetum | 0.19 | 0.38 |
| Symphoricarpos albus | Pyrolo-Abietetum | 0.19 | 0.37 |
| Taxus baccata | Pyrolo-Abietetum | 0.19 | 0.38 |
| Tilia cordata | Pyrolo-Abietetum | 0.19 | 0.38 |
| Valeriana dioica | Pyrolo-Abietetum | 0.19 | 0.38 |
| Valeriana officinalis | Pyrolo-Abietetum | 0.19 | 0.37 |
| Verbascum phlomoides | Pyrolo-Abietetum | 0.19 | 0.36 |
| Vicia cracca | Pyrolo-Abietetum | 0.19 | 0.39 |
| Vinca minor | Pyrolo-Abietetum | 0.19 | 0.38 |
| Knautia dipsacifolia | Pyrolo-Abietetum | 0.17 | 0.42 |
| Taraxacum officinale agg. | Pyrolo-Abietetum | 0.17 | 0.78 |
| Heracleum sphondylium | Pyrolo-Abietetum | 0.16 | 0.47 |
| Chaerophyllum hirsutum | Pyrolo-Abietetum | 0.16 | 0.50 |
| Salix aurita | Pyrolo-Abietetum | 0.16 | 0.66 |
| Poa trivialis | Pyrolo-Abietetum | 0.15 | 0.59 |
| Phyteuma spicatum | Pyrolo-Abietetum | 0.15 | 0.61 |
| Melampyrum sp. | Pyrolo-Abietetum | 0.15 | 0.75 |
| Cardamine pratensis | Pyrolo-Abietetum | 0.14 | 0.74 |
| Epilobium parviflorum | Pyrolo-Abietetum | 0.14 | 0.73 |


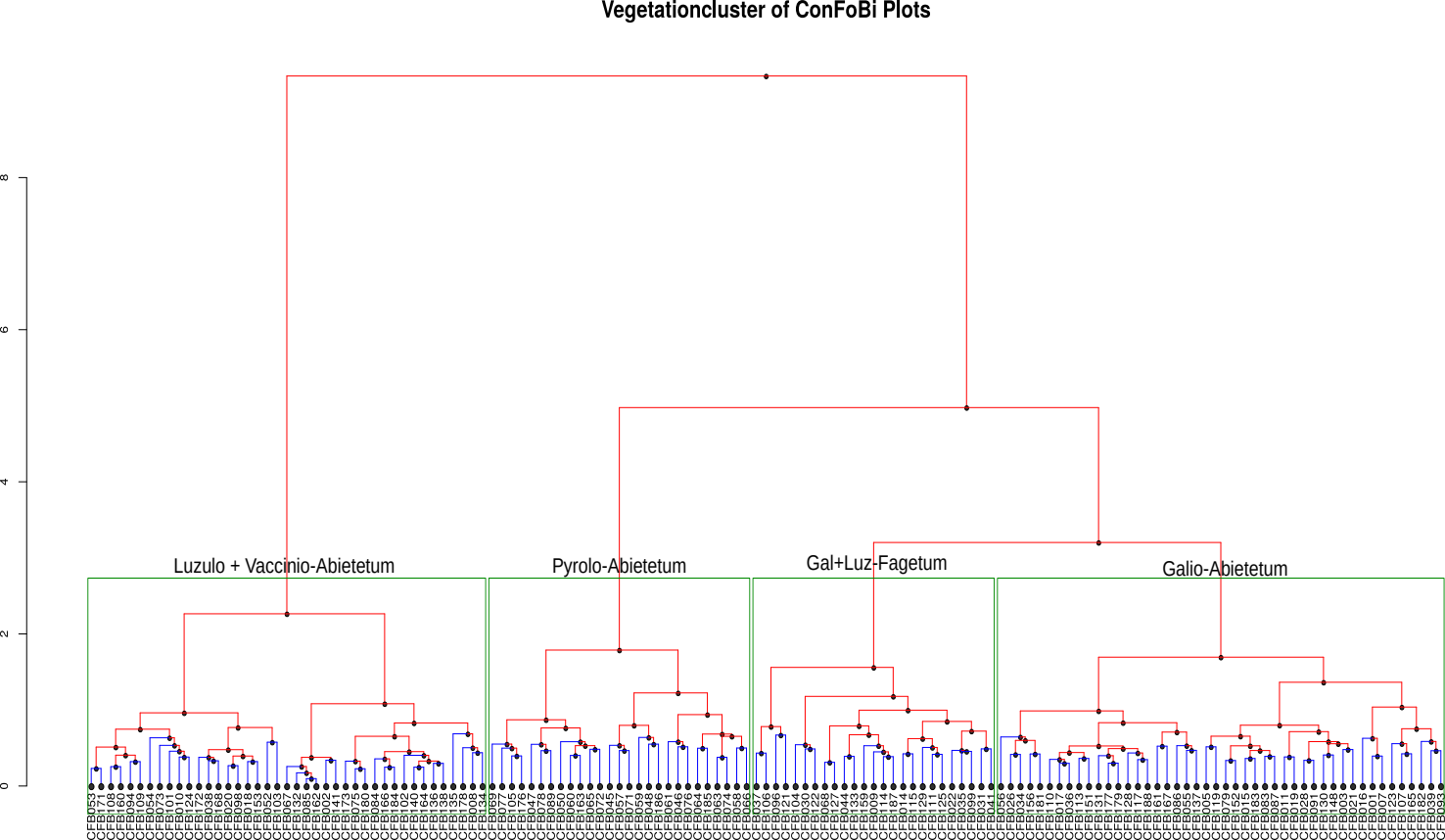


Figure S3: Classification of forest communities. Detailed information of the classification process can be found in the main manuscript.


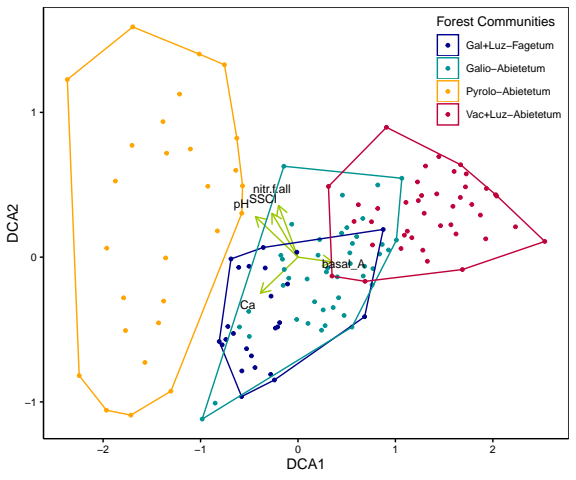


Figure S4: Detrended correspondence analysis of the plots with the different forest communities and the direction of significant predictors (significance level < 0.05). Cover estimates of herb species (determined on the aggregate level) was used.

In the following we describe in how the forest communities differ. Table S2 shows that the communities significantly differ in species richness (also shown in Fig. S5a). This means that the forest communities inhabit initially different numbers of species. As the major aim is to investigate the effects of heterogeneity on species richness, we have to account for these initial species richness differences. As we are interested only on the effects of heterogeneity particularly, we used the forest community as random effect in mixed effect models. Other relationships of the forest communities which showed significant effects are shown in Fig. S5.

Table S2: Mean and standard deviation for the different forest communities. Letters indicates significant differences (p < 0.05) analyzed by a Tukey–test. Letters indicate: A = Gal+Luz-Fagetum, B = Galio-Abietetum, C = Pyrolo-Abietetum, D = Vac+Luz-Abietetum.

| Measure | Gal+Luz-Fagetum | Galio-Abietetum | Pyrolo-Abietetum | Vac+Luz-Abietetum |
| --- | --- | --- | --- | --- |
| DLI (%) | 12.53 ± 7.09 | 16.25 ± 7.74 | 15.04 ± 8.99 | 16.99 ± 7.33 |
| DLI_cv(%) | 55.63 ± 26.34 | 41.43 ± 19.35 | 56.23 ± 32.89 | 43.29 ± 30.96 |
| pH | 3.7^CD^ ± 0.26 | 3.54^CD^ ± 0.26 | 4.96^ABD^ ± 0.75 | 3.11^ABC^ ± 0.38 |
| pH_cv (%) | 4.27^BCD^ ± 2.00 | 8.46^A^ ± 5.55 | 9.73^A^ ± 5.41 | 8.09^A^ ± 2.98 |
| C:N-ratio (%) | 17.26^D^ ± 1.66 | 18.19^D^ ± 2 | 17.46^D^ ± 5.01 | 20.6^ABC^ ± 3.45 |
| C:N-ratio_cv (%) | 9.86 ± 5.12 | 8.9^C^ ± 4.31 | 12.73^BD^ ± 7.34 | 9.32^C^ ± 3.66 |
| BA (m_2_/ha) | 34.01 ± 14.54 | 34.9 ± 9.33 | 31.16 ± 9 | 34.54 ± 7.73 |
| DBHcv(%) | 50.12 ± 14.86 | 49.26 ± 18.82 | 58.25 ± 17.44 | 53.4 ± 16.68 |
| SSCI | 4.42 ± 1.62 | 3.71^C^ ± 1.07 | 4.95^B^ ± 1.74 | 4.35 ± 1.82 |
| TRI | 0.46^B^ ± 0.14 | 0.59^A^ ± 0.15 | 0.56 ± 0.15 | 0.56 ± 0.16 |
| SR_herb | 29.08^CD^ ± 12.15 | 35.54^CD^ ± 9.06 | 45.85^ABD^ ± 12.77 | 18.34^ABC^ ± 10.45 |
| Altitude (m) | 633.25^BD^ ± 142.82 | 930.44^AC^ ± 170.48 | 682.4^BD^± 92.09 | 892.85^CA^ ± 133.22 |


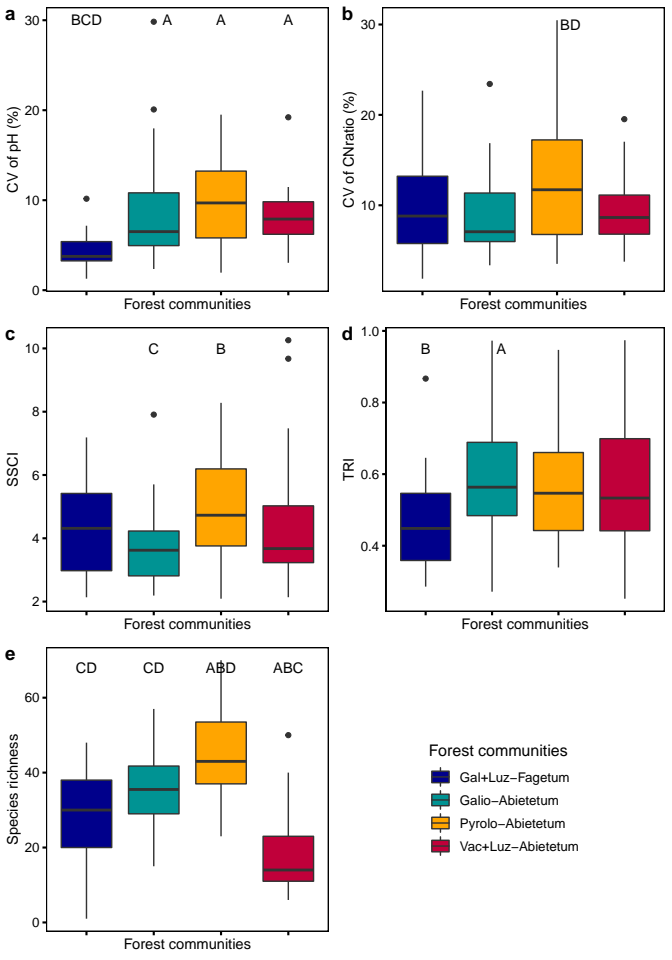


Figure S5: Box plots of pH_cv (%), C:N-ratio_cv, SSCI, TRI and species richness. Only plots are shown where the communities showed significant differences in the respective variable (p < 0.05) and are a predictor of interest. Letters indicates significant differences (p < 0.05) analyzed by a Tukey–test. Letters indicate: A = Gal+Luz-Fagetum, B = Galio-Abietetum, C = Pyrolo-Abietetum, D = Vac+Luz-Abietetum.

# Model output of significant best model candidates

Table S3: H1, model output. Effect of BA on light heterogeneity. Model structure: lm(DLI_cv ~ BA + DLI)

|  | Estimate | Std. Error | t value | Pr(>\|t\|) |
| --- | --- | --- | --- | --- |
| (Intercept) | 106.4808 | 10.1557 | 10.4848 | 0.0000 |
| basal_A | -1.0816 | 0.2400 | -4.5075 | 0.0000 |
| DLI | -1.4564 | 0.2945 | -4.9455 | 0.0000 |

Table S4: H1, linear model output. Effect of DBHcv on light heterogeneity. Model structure: lm(DLI_cv ~ DBHcv + DLI)

|  | Estimate | Std. Error | t value | Pr(>\|t\|) |
| --- | --- | --- | --- | --- |
| (Intercept) | 22.5966 | 9.2316 | 2.4477 | 0.0160 |
| DBHcv | 0.7346 | 0.1297 | 5.6627 | 0.0000 |
| DLI | -0.9089 | 0.2862 | -3.1759 | 0.0019 |

Table S5: H1, linear model output. Effect of SSCI on light heterogeneity. Model structure: lm(DLI_cv ~ SSCI + DLI)

|  | Estimate | Std. Error | t value | Pr(>\|t\|) |
| --- | --- | --- | --- | --- |
| (Intercept) | 36.1722 | 8.5958 | 4.2081 | 0.0001 |
| DLI | -1.0242 | 0.2972 | -3.4466 | 0.0008 |
| SSCI | 6.2659 | 1.4066 | 4.4548 | 0.0000 |

Table S6: H1, linear model output. Effect of TRI on light heterogeneity. Model structure: lm(DLI_cv ~ TRI + DLI)

|  | Estimate | Std. Error | t value | Pr(>\|t\|) |
| --- | --- | --- | --- | --- |
| (Intercept) | 43.1115 | 10.5992 | 4.0674 | 0.0001 |
| DLI | -1.2638 | 0.3185 | -3.9675 | 0.0001 |
| TRI | 42.2496 | 16.9105 | 2.4984 | 0.0141 |

Table S7: H1, generalized linear mixed effect model output. Effect of DBHcv on pH heterogeneity. Model structure: glmer(pH_cv ~ DBHcv + DLI + DLI_cv + pH + DBHcv:DLI + (1|f. community), family = “gaussian”(link=”log“)

|  | Estimate | Std. Error | t value | Pr(>\|t\|) |
| --- | --- | --- | --- | --- |
| (Intercept) | 1.9638 | 0.1690 | 11.6220 | 0.0000 |
| DBHcv | 0.1457 | 0.0504 | 2.8888 | 0.0039 |
| DLI | 0.0644 | 0.0462 | 1.3953 | 0.1629 |
| DLI_cv | -0.1191 | 0.0620 | -1.9200 | 0.0549 |
| pH | -0.1610 | 0.0790 | -2.0374 | 0.0416 |
| DBHcv:DLI | -0.1495 | 0.0364 | -4.1086 | 0.0000 |

Table S8: H1, generalized linear mixed effect model output. Effect of TRI on pH heterogeneity. Model structure: glmer(pH_cv ~ TRI + DLI + pH + TRI:DLI + (1|f. community), family = ”gaussian“(link=”log“)

|  | Estimate | Std. Error | t value | Pr(>\|t\|) |
| --- | --- | --- | --- | --- |
| (Intercept) | 2.0761 | 0.0533 | 38.9735 | 0.0000 |
| DLI | 0.1391 | 0.0488 | 2.8534 | 0.0043 |
| pH | 0.0086 | 0.0476 | 0.1806 | 0.8567 |
| TRI | 0.0801 | 0.0529 | 1.5154 | 0.1297 |
| DLI:TRI | -0.1643 | 0.0470 | -3.4961 | 0.0005 |

Table S9: H1, generalized linear mixed effect model output. Effect of SSCI on C:N-ratio heterogeneity. Model structure: glmer(C:N-ratio_cv ~ CNratio + DLI + DLI_cv + SSCI + CNratio:DLI + CNratio:SSCI + DLI_cv:SSCI + DLI:SSCI + (1|f. community), family = ”gaussian“(link=”log“)

|  | Estimate | Std. Error | t value | Pr(>\|t\|) |
| --- | --- | --- | --- | --- |
| (Intercept) | 2.3375 | 0.0497 | 47.0407 | 0.0000 |
| CNratio | 0.1334 | 0.0412 | 3.2368 | 0.0012 |
| DLI | -0.0482 | 0.0458 | -1.0513 | 0.2931 |
| DLI_cv | -0.0113 | 0.0522 | -0.2157 | 0.8292 |
| SSCI | 0.0092 | 0.0494 | 0.1861 | 0.8524 |
| CNratio:DLI | -0.0623 | 0.0435 | -1.4332 | 0.1518 |
| CNratio:SSCI | 0.0728 | 0.0432 | 1.6842 | 0.0922 |
| DLI_cv:SSCI | -0.1230 | 0.0465 | -2.6430 | 0.0082 |
| DLI:SSCI | -0.0718 | 0.0424 | -1.6936 | 0.0903 |

Table S10: H1, generalized linear mixed effect model output. Effect of SSCI on C:N-ratio heterogeneity. Model structure: glmer(C:N-ratio_cv ~ CNratio + DLI_cv + TRI + CNratio:DLI_cv + CNratio:TRI + DLI_cv:TRI + CNratio:DLI_cv:TRI + (1|f. community), family = ”gaussian“(link=”log“)

|  | Estimate | Std. Error | t value | Pr(>\|t\|) |
| --- | --- | --- | --- | --- |
| (Intercept) | 2.3537 | 0.1133 | 20.7716 | 0.0000 |
| CNratio | 0.1828 | 0.0421 | 4.3443 | 0.0000 |
| DLI_cv | -0.0198 | 0.0494 | -0.3998 | 0.6893 |
| TRI | 0.1437 | 0.0490 | 2.9330 | 0.0034 |
| CNratio:DLI_cv | -0.0416 | 0.0419 | -0.9936 | 0.3204 |
| CNratio:TRI | 0.0674 | 0.0531 | 1.2686 | 0.2046 |
| DLI_cv:TRI | 0.0318 | 0.0485 | 0.6559 | 0.5119 |
| CNratio:DLI_cv:TRI | -0.0907 | 0.0326 | -2.7801 | 0.0054 |

Table S11: H2, generalized linear mixed effect model output. Effect of selected heterogeneity variables on species richness. First model selection run. Final model structure: glmer.nb(SR_herb_agg ~ DLI_cv + DLI + (1|f. community))

|  | Estimate | Std. Error | z value | Pr(>\|t\|) |
| --- | --- | --- | --- | --- |
| (Intercept) | 3.1260 | 0.1862 | 16.7884 | 0.0000 |
| DLI | 0.0698 | 0.0336 | 2.0789 | 0.0376 |
| DLI_cv | 0.0840 | 0.0345 | 2.4377 | 0.0148 |

Table S12: H2, generalized linear mixed effect model output. Effect of selected heterogeneity variables on species richness. Final model structure: glmer.nb(SR_herb_agg ~ CN-ratio_cv * DLI_cv + DLI + (1|f. community))

|  | Estimate | Std. Error | z value | Pr(>\|t\|) |
| --- | --- | --- | --- | --- |
| (Intercept) | 3.1124 | 0.1766 | 17.6271 | 0.0000 |
| CNratio | -0.0521 | 0.0335 | -1.5559 | 0.1197 |
| CNratio_cv | 0.0459 | 0.0318 | 1.4420 | 0.1493 |
| DLI | 0.0733 | 0.0325 | 2.2553 | 0.0241 |
| DLI_cv | 0.0877 | 0.0334 | 2.6211 | 0.0088 |
| CNratio_cv:DLI_cv | 0.0849 | 0.0342 | 2.4793 | 0.0132 |

# Abreviations:

BA = Basal area
DBH = Diameter of breast hight
DBHcv = Coefficient of variation of diameter of breast hight
DLI = Diffuse light index
DLI_cv = Coefficient of variation of diffuse light index
f. community = forest community
SSCI = Stand structural complexity index (Ehbrecht et al. 2017)
TRI = Terrainian ruggednes index
Gal+Luz-Fagetum = Galio + Luzulo Fagetum
Vac+Luz-Abietetum = Vaccino + Luzulo Abietetum
